# Trace Element Analysis by ICP-MS and Chemometric Approach in Some *Euphorbia *Species: Potential to become a Biomonitor

**DOI:** 10.22037/ijpr.2019.1100875

**Published:** 2019

**Authors:** Ismail Yener, Hamdi Temel, Ozge Tokul-Olmez, Mehmet Firat, Elif Varhan Oral, Mehmet Akdeniz, Kerem Senturk, Erhan Kaplaner, Mehmet Ozturk, Abdulselam Ertaş

**Affiliations:** a *Department of Analytical Chemistry, Faculty of Pharmacy, Dicle University, TR-21280 Diyarbakir, Turkey. *; b *Department of Pharmaceutical Chemistry, Faculty of Pharmacy,* *Dicle University, TR-21280 Diyarbakir, Turkey.*; c *Department of Chemistry, Faculty Science, Mugla Sıtkı Koçman University, TR-48121 Mugla, Turkey.*; d *Department of Biology, Faculty of Education, Yüzüncü Yıl University, TR-65080 Van, Turkey.*; e *The Council of Forensic Medicine, Ministry of Justice, Diyarbakir, 21100, Turkey.*; f *Department of Pharmaceutical Toxicology, Faculty of Pharmacy, Dicle University, TR-21280 Diyarbakir, Turkey. *; g *Department of Pharmacognosy, Faculty of Pharmacy, Dicle University, TR-21280 Diyarbakir, Turkey.*

**Keywords:** Euphorbia, Trace Element, ICP-MS, Ornamental Plants, Chemometric Approach

## Abstract

In this study, the branch, leaves, flowers, roots and mixed parts of different nine *Euphorbia* species were analyzed for their trace element contents by using ICP-MS. The samples were digested by concentrated nitric acid and hydrogen peroxide in a microwave by ICP-MS before the analysis. The accuracy and precision of the method was evaluated by CRM 1573a Tomato Leaves. Trace element contents accumulated in different parts of each sample were contrasted. Minitab Statistical Software Inc., programme was used for the multivariate analysis of 12 toxic metals of seeds, roots, branches, leaves, flowers, and mixed parts of *Euphorbia* species collected from Diyarbakir, Kayseri, Malatya, Mardin, Trabzon, and Van cities.When the studied *Euphorbia* species are compared in terms of their metal contents; V, Tl, Cr, and Ni metals in *E. eriophora*, Ba in *E. aleppica*, As and Co metals in *E. segıieriana*, Ag and Se metals in *E. craspedia*, Cu and Cd metals in *E. fistulosa*, Cs and Pb metals in *E. grisophylla*, Zn in *E. macroclada* and also Rb and Sr metals in *E. denticulata *were determined higher. It was determined that the studied species accumulated some metals at highly amounts especially in the root and leaf parts. In general, it can be said that *Euphorbia* species have high potential to become a biomonitor. For this reason, it can be predicted that these species will be used as ornamental plants in landscape architecture due to both their toxic metals retention properties and their beautiful appearance.

## Introduction

*Euphorbia* L. (Euphorbiaceae), with over 2000 species (1), is the second-largest genus of flowering plants, outsized only by *Astragalus *L. (2). The genus occupies a wide array of habitats including deserts, coastal dunes, steppe grasslands, shrublands and forests, riparian areas, rocky slopes, and cliffs at elevations ranging from sea level to over 4000 m (3). *Euphorbia* is represented in Turkey by two subgenera (subg. *Chamaesyce*, subg. *Esula*) with a total of 111 taxa (except for cultivated ones) (4). *Euphorbia macroclada* (Spurge): Plantlatexis used externally for treatment of warts (5). *E. macroclada*is only used for feeding camels, not other animals. Local inhabitants know that it is poisonous. *E. denticulata*; the root part of the plant and also the drained milk of the plant that flow in the plant are used to cease diarrhea and abdominal pain, *E. macrocarpa; *the gained milk that flows through the plant is spread to the wounded region on the body surface, *E. virgata; *the milk that gained from the plant is eaten by dropping a few drops of this milk to the sugar, this mixture is used in constipation treatment, and also its flowers are used for eczama treatment (6). *E. gripsophylla*; the milk that drained from the plant is drunk by mixing 1-2 drops of milk to the water, this mixture is used in constipation treatment too (7).

A literature survey of the genus showed that many of its constituents are highly bioactive in phytochemical analyses (8-10). Many different parts of the *Euphorbia* species like roots, seeds, latex, stem, stem barks, leaves, and whole plants have been studied. Moreover, it is found that the plants in the Euphorbiaceae family are well known for the chemical diversity of their isoprenoid constituents (11). The major constituents of the genus are diterpenoids (12,13). Many biological activities of the constituents of the* Euphorbia *species have been reported for a decade (10,13-15).

Trace elements play a significant role in the formation of chemical constituents in plants. It is known that twenty-three elements have physiological activities in mammals (16). Some metals such as zinc, iron, copper, chromium, and cobalt are necessary at certain levels and they are toxic in high concentrations. On the other hand, some other metals; namely, mercury, lead and cadmium, are toxic even at low concentrations and have been known not to have useful properties. Determining the metal ion compositions of plantssupport their medicinal, nutrient and/or toxic properties (17).

Trace elements have important roles in plant metabolism and biosynthesesas cofactors for the enzymes.Medicinal plants are widely used in the treatment of human diseases and pain relief, due to their low adverse effects (18). Some medicinal plants and their mixtures may pose health risks, owing to toxic element contains. The contamination may become from the environmental pollution.For example, high levels of arsenic can result from the use of pesticides and fertilizers. Human beings need metallic and nonmetallic elements, within the permitted limits, for growth and health. The plants are an important medium for trace elements to transit from the soil to human beings. Accordingly, the quality controls of these medicinal plants are important in terms of the trace element content (19).

Graphite furnace atomic absorption spectrometry (GF-AAS) (20), flame atomic absorption spectrometry (F-AAS) (18,21), inductivelycoupled plasma optical emission spectrometry (ICP-OES) (21,22), inductively coupled plasma–massspectrometry ICP-MS (23), instrumental neutron activation analysis (INNA) (24,25) or X-ray fluorescence spectrometry (XRF) (26) techniques are used to determine the trace element contents of the medicinal plants. ICP-MS takes part out of these techniques, and also this technique is more effective in the determination of multiple elements at trace levels due to its high sensitivity, precision, and large linear dynamic range.

The most common used chemometric techniques are Principal Component Analysis (PCA) and Hierarchical Clustering Analysis (HCA). PCA seeks for an answer about the relationship between the samples and the interaction between variables. Clustering technique (CA), however, provides information regarding the classification (characterization) of the samples. These techniques reveal relationships of classification and the predictions that cannot be considered as “ordinary results” (27).

In this study, we aimed to determine toxic and nutrient elements concentrations of roots, stems, leaves, flowers, and mixed samples of nine *Euphorbia *species, collected from Diyarbakir, Kayseri, Malatya, Mardin, Trabzon and Van cities by using ICP-MS. Also, *E. macroclada* species were compared each other by collected from 4 different localities.In addition, classification of trace metal components and evaluation of differences between sections were done by using PCA and HCA methods. 

## Experimental

*The plant materials*

We collected the whole plants of *Euphorbia *species from southeast of Turkey in July 2015 by Dr. A. Ertaş (Department of Pharmacognosy, Faculty of Pharmacy, Dicle University) and Mehmet Fırat (Department of Biology, Faculty of Education, Yuzuncu Yil University) andcollected plants/species were identified by Mehmet Fırat. Voucher specimens were strored in the Herbarium of Yuzuncu Yil University 

(Table 1).

*Instruments*

Agilent 7700X model ICP-MS was used for the determination of Ag, As, Cd, Co, Cr, Cu, Ni, Pb, Se, Tl, V, and Zn in the samples. The operating conditions for the ICP-MS were shown in Table 2**. **Digestion procedure of the samples prior to analysis was carried out in a Milestone Start D Brand microwave oven equipped and then the samples transferred to polytetrafluorethylene (PTFE) vessels.

*Reagents and solutions*

For analytical purity ultrapure nitric acid (Merck), ultrapure hydrogen peroxide (Merck). And 18.2 MΩ deionized distilled water was used in all experiments. In the ICP-MS measurements, a high purty solution from ^6^Li, ^45^Sc, ^72^Ge, ^115^In, and ^209^Bi were used as the mix internal standard (200 μg L^-1^). After diluted at 10 mg L^-1^mix standard in the concentration range of 0-100 μg L^-1^, the calibration graphics were prepared for As, Ag, Ba, Be, Cd, Co, Cr, Cu, Cs, Hg, Li, Ni, Pb, Rb, Se, Sr, Tl, U, V, and Zn metals. The accuracy and precision of the method were evaluated by using a CRM NIST 1573a Tomato Leaves (National Institute of Standards and Technology, NIST, Gaithersburg, MD, USA) Certified Standard Reference Material. 

*Sample preparation*

The Samples were divided into groups as roots, leaves, stems, flowers and mixed, washed by tap water first and deionized water secondly, and dried at 70 °C for 48 h (19). The dried samples were powdered by using a blender. Generally, the abbreviations of all species were given in Table 1. When the species names were abbreviated, they were formed as the first letters of the genus, and then species and also if any of the collection localities were exist they specified, finally attached which part was used. When the studied species were separated into parts, they were sembolized as Root (R), branch (B), leaf (L), flower (F), seed (S) and mixture of them (the mix) (M). The dried samples were then pulverized by a blender. About 200 mg of the pulverized samples were accurately weighed into polytetrafluorethylene (PTFE) digestion vessels and 6 mL HNO_3 _and 2 mL H_2_O_2_ were added; then, they were digested in a microwave oven. The digested samples were taken into 25 mL volumetric flasks and filled by deionized water. Blank tests were carried out as three independent experiments in the sameway. The certified standard reference material CRM 1573a Tomato Leaves (National Institute of Standards and Technology, NIST, Gaithersburg, MD, USA) was applied for the same digestion method mentioned above.

*Method validation*

Linear range, regression correlation coefficient (R), Limits of detection (LOD) and limits of quantification (LOQ) values regarding the calibration curve drawn for twenty elements under optimized working conditions are presented in Table 3. The fact that R value appears to be higher than 0.99 indicates that linearity is acceptable. LOD and limits LOQ (LOD = 3.σ and LOQ = 10.σ) are calculated for twenty metals by means of three independent analysis.

Findings regarding the certified standard reference material (CRM NIST 1573a Tomato Leaves (National Institute of Standards and Technology, Gaithersburg, MD, USA) analyzed to evaluate the accuracy of the method are presented in Table 4. 

*The chemometric analysis*

The chemometric analyses of the metal contents of roots, leaves, stems, flowers, and mixed parts of twelve *Euphorbia* species are carried out using Principal Component Analysis (PCA) and Hierarchical Clustering Analysis (HCA), which are multivariate data analysis methods. Both methods for clustering and classification are mainly based upon the principal component analysis. 

PCA reduces multiple variables into a set of fewer components created by their linear combinations by hindering correlations between those examined variables. PCA-based methods can classify the samples by clustering into various groups. Hierarchical Clustering Analysis (HCA) classifies the samples in a given data set and defines those data according to their similarities. HCA can be applied directly to the original variables, as well as possible to be applied to the results obtained from PCA, in case of existing too many variables. In this study, HCA applied to the results of the analysis of trace metal components, the measurement is based on the Euclidean distance. 

The Ward’s method is used as a clustering method. In this context, all classification and clustering analyses for *Euphorbia* species were carried out using MINITAB Statistical Software.

*Statistical method*

All statistical calculations were done using Minitab 16.2.1. statistical software (MINITAB Inc, 2010). Multivariate analysis of 12 toxic metals of *Euphorbia *species was performed using Principle component analysis (PCA) and hierarchical clustering analysis (HCA) techniques. 

The chemometrics analysis was applied to discriminate and of roots, leaves, stems, flowers and mixed parts and geographic origins of 9 *Euphorbia* species according to 12 heavy metal contents. The main goal while performing PCA and HCA methods to whole dataset was to examine similarities between the *Euphorbia* samples and also the metal contents.” was added to the Statistical Data Processing part.

## Results and Discussion

*Concentrations of elements in Euphorbiaspecies*

Leaves, flowers, roots, stems, and mixed parts of five *Euphorbia* species were examined in this study for their metal contents, and the results were presented in Tables 5-7. 

Countries in different parts of the world were determinated toxic metal limit levels differently for the medicinal plants. In the raw medicinal herbal materials toxicity limits for lead, arsenic, chromium, and cadmium were reported as 10, 5, 2 ve 0.3 ppm, respectively. For finished herbal products or dietary supplements, the toxicity limits were determinated differently. The toxicity limits for lead, chromium, and mercury is 0.02 mg/day, for arsenic is 0.01 mg/day and for cadmium is 0.006 mg/day for a 60 kg person (28).

Cobalt (Co) is a micronutrient element for plants. In plants, Co complex is found in the form of vitamin B12 (29). It is seen that Co concentrations of studied species range from 0.106 to 1.699 mg kg^-1^. And also Cobalt concentrations seems to be lower than the accepted value of 1.0 mg kg^-1^ (30) except 4 roots (EGVR, ESDR, EFDR, EMVR), EMVL and all parts of *E. seguieriana *subsp.* seguieriana*. At the same time, the all parts of all *Euphoria* species studied are found to be lower than the 15 mg kg^-1^ concentration (30). When the all species studied are examined, the highest amount of Co was found in the root parts of the concentration in all species, except *E. seguieriana* subsp. *seguieriana* and *E. macroclada* which collected from Diyarbakır and Trabzon. Additionally, the Co concentrations accumulated in the roots of all species are ranked in the order of EMVR > ESDR > EGVR > EFDR > ECMR > EADR > EMMR > EMDR > EEDR > EDKR > EMTR are determinated too. In the study of Pehlivanlı *et al.,* the amount of Co in *Euphorbia macroclada* plant has been determined as 1 ppm (31); however, the amount of Co in root and shoot part of *Euphorbia macroclada Boiss* has been found as 1.07-6.65 and 0.28-1.13 mg kg^-1^, respectively, in the study of Sasmaz and Yaman (30).

Nickel (Ni) is recognized as an essential micronutrient for living organisms. Nickel is a component of the enzyme urease, and essential for its function and good health in animals (29). It is determinated that Ni concentrations of the studied species range from 0.344 to 12.209 mg kg^-1^ and also Ni concentrations are found lower than the normal accepted value of 5.0 mg kg^-1^ (30) for dry plants except six different roots (ECMR, EADR, EGVR, ESDR, EFDR, EMVR) and five different parts (EMML, EMDS, ECMS, ESDL, ESDF)*. *At the same time, all parts of the species among studied species and parts, except for the ESDR and EFDR parts, containes less than 10 mg kg^-1^concentration of Ni (30), which is accepted excessive/toxic for Ni. It is determined that the maximum concentration of Ni is collected in the root parts of the all species studied except EMML, EMDS, and EMTS. And also the Ni concentrations accumulate in the roots of all species are ranked in the order of ESDR > EFDR > EMVR > EGVR > ECMR > EADR > EMDR > EEDR > EMMR > EDKR > EMTR are determinated. In the study of Pehlivanlı *et al*., the amount of Ni has been found in *Euphorbia macroclada* plant as 3.50 ppm (31); however, the amount of Ni in root and shoot part of *Euphorbia macroclada Boiss* has been found as 2.73-13.1 and 0.97-2.23 mg kg^-1^, respectively, in the study of Sasmaz and Yaman.(30). In the study of Ugulu *et al*, the amount of Ni has been found as 0,340 μg g^-1^ in *Euphorbia sp.* which was collected from 1000 m height of Murat Mountain. Also, at height of 1.600 m they have been found as 1.976 μg g^-1^ in *Euphorbia anacampseros Boiss subsp. anacampseros* plant and 3.326 μg g^-1^ in *Euphorbia rigida M. bieb.* plant.

Chromium (Cr), another essential element, acts as a co-factor in insulin synthesis and cholesterol synthesis. In this study, it is determinated that Cr concentrations of the studied species range from 0.213 to 12.913 mg kg^-1^ and also Cr concentrations are found higher than the normal accepted value of 0.5 mg kg^-1^ (30) value except EDKS, EDKB, EDKL, EDKF, EGVS, ESDS, ESDF, EMDS, EMVS, EMTS, EMTF, and EMTL parts. At the same time all parts of the species among the studied species and parts, except for the EGVR, ESDR, EFDR, ECMR, EDKR, EADR, EEDR, EMMR, EMDR, and EMVR parts, containe less than 5 mg kg^-1^concentration of Cr (30) , which are accepted excessive/toxic for Cr. (28). As it can be seen from Tables 5, 6, and 7, it is determined that the Cr concentrations considered as excessive / toxic for all species are found in the root parts and that the accumulation of Cr was the most especially in the root parts. Additionally, it is determinated that the Cr concentrations accumulating in the roots of all species are ranked in the order of EFDR > EMVR > ESDR > EGVR > EMMR > EADR > ECMR > EEDR > EDKR > EMDR > EMTR. In the study of Sasmaz and Yaman, the amount of Cr in root and shoot part of *Euphorbia macroclada Boiss* has been found as 0.86-8.88 and 042-0.96 mg kg^-1^, respectively.

Arsenic (As) can be widely found in foods, usually medical plants, vegetables, legumes and grains are an important pathway for the intake of arsenic for humans. But because of its toxicity it is important to monitor the arsenic in the food consumed by humans (32). Arsenic (As) concentrations of the studied species range from 0.011 to 1.203 mg kg^-1^ and also all As concentrations measured from studied species are found lower than the normal accepted value of 5 mg kg^-1^ whose value is determinated from World of Health Organisation (WHO) for raw medicinal plant samples (28, 30). As it can be seen from Tables 5, 6 and 7, Arsenic (As) accumulates in root parts mostly for the all species studied. And it is determinated that the As concentrations accumulating in the roots of all species are ranked in the order of ESDR > EDKR > EGVR > EMVR > ECMR > EMTR > EFDR > EADR > EEDR > EMMR > EMDR.

Cadmium (Cd) concentrations of the studied species ranged from 0.005 to 0.479 mg kg^-1^ and also Cd concentrations of the all studied species except EGVR, ESDR, ECMR, and ECMB were found lower than the normal accepted value of 0.3 mg kg^-1^ that was determinated from WHO for raw medicinal plant samples. As it can be seen from Tables 5, 6 and 7, Cadmium (Cd) accumulates in root parts mostly for all species studied. And it was determinated that the Cd concentrations accumulated in the roots of all species are ranked in the order of EGVR> ECMR > ESDR > EFDR > EMTR > EMMR > EEDR > EDKR > EADR = EMDR > EMVR. In the study of Pehlivanlı *et al*. , the amount of As has been found as 0.20 ppm in *Euphorbia macroclada* plant; and the World Plant average is defined as 0,04 ppm for As (31, 33).

Lead (Pb), which is an environmental pollutant, accumulates in bones in humans. Also excessive Pb exposure leads to behavioral disturbance and mental retardation by preventing intelligence development (34-36). Lead (Pb) concentrations of the studied species ranged from 0.071 to 6.813 mg kg^-1^ and also Pb concentrations of the all studied species were found lower than the normal accepted value of 10 mg kg^-1^ that was determinated from WHO for raw medicinal plant samples (28, 37). As it can be seen from Tables 5, 6 and 7, Cadmium (Cd) accumulates in root parts mostly for the all species studied except *E. denticulata* and *E. macroclada* (collected from Diyarbakır and Trabzon). Additionally, it is determinated that the Pb concentrations accumulates in the roots of the all species are ranked in the order of EGVR > ESDR > EFDR > ECMR > EADR > EDKR > EEDR. In the study of Pehlivanlı *et al*. , the amount of Pb has been found as 0.70 ppm in *Euphorbia macroclada p*lant; however the World Plant average is defined as 15,60 ppm for Pb (31, 33). Copper (Cu) is an essential heavy metal for higher plants and algae, particularly for phosynthesis (29, 38). Cu is a constituent of primaryelectron donor in photosystem I, the copper protein plastocyanin. Because Cu can readily gain and lose electron, it is a cofactor of oxidases, mono-and di oxygenase (e.g. amine oxidase, ammonia monoxidase, ceruloplasmin, lysyl oxidase) and of enzymes involved in the elimination of superoxide radicals (e.g. superoxide dismutase and ascorbate oxidase). Several enzymes contain copper, such as ascarbonic anhydrase, alcohol dehydrogenase, superoxide dismutase, and RNA polymerase. It is also required to maintain the integrity of ribosome. It takes part in the formation of carbonhydrates, and catalyzes the oxidation processes in the plants. Line also provides a structural role in many transcription factors and is a cofactor of RNA polymerase (29). But it is toxic to plants at high concentrations (39). It is harmful to human health when taken exremely by humans. Furthermore, it can decrease the hypertension and infertility effect of lead (40). Copper (Cu) concentrations of the studied species range from 2.297 to 44.488 mg kg^-1^. Pehlivanlı et al., have found the amount of Cu as 11,60 ppm in *Euphorbia macroclada* plant; and the World Plant average is defined as 4,98 ppm for Pb (31, 33).

Zinc (Zn) also plays essential metabolic roles in the plant, of which the most significant is its activity as acomponent of a variety of enzymes, such as dehydrogenase, proteinases, peptidases, and phospohydrolases (41). Shkolnik and Leringrad (1974) indicates that the basic Zn functions in plants are related to the metabolisms of carbonhydrates, proteins, and phosphate and also to auxin, RNA, and ribosome synthesis (42). Zinc (Zn) concentrations of the studied species range from 10.348 to 79.547 mg kg^-1^. Also, Zn element accumulates mainly in the leaf, seed, root and flower of the studied species. Zinc is one of the essential element for plant growth, and also it is found higher in *Euphorbia* samples. Similar result has been obtained in the study of the purple coneflower (*Echinacea purpurea*) (43, 44). In the study of Pehlivanlı *et al*., the amount of Zn has been found in *Euphorbia macroclada* plant as 27,00 ppm (31); however, in the study of Ugulu *et al*, the amount of Zn has been found as 0,556 μg g^-1^ in *Euphorbia sp.* collecting from 1000 m height of Murat Mountain. At height of 1,600 m they have been found as 0,846 μg g^-1^ in *Euphorbia anacampseros Boiss subsp. anacampseros* plant and 0,833 μg g^-1^ in *Euphorbia rigida M. bieb. *plant (45).

Thallium (Tl) is more toxic than the other known toxic metals such as mercury, lead and cadmium and Thallium that can be harmful to living organisms even at very low levels (46, 47). Tl intake at 20-60 mgkg^-1^ body weight can be fatal within one week. Fortunately, Tl level is very low in environmental samples such as soil and water. In our study it was determinated that thallium (Tl) concentrations of the studied species ranged from 0.0006 to 0.159 mg kg^-1^. As it can be seen from Tables 5, 6, and 7, Thallium (Tl) accumulates in root parts mostly for the all species studied except *E. Macroclada* collected from Trabzon. Additionally, it was determinated that the Tl concentrations accumulated in the roots of the studied species are ranked in the order of EGVR > EMVR > ESDR > EMMR > ECMR > EFDR > EDKR > EADR > EEDR > EMTR > EMDR. In the study of Pehlivanlı *et al.* , the amount of Tl has been fond as 0,30 ppm in *Euphorbia macroclada* plant; and the World Plant average is defined as 0,01 ppm for Tl (31, 33).

Selenium is an another essential element for humans and animals. Selenium exhibits antioxidant properties. Also selenium, a toxic trace element, and has a protective effect against cancer and heart disorders (48). In this study selenium (Se) concentrations of the studied species range from 0.307 to 2.430 mg kg^-1^. Se amounts are observed highest in the flower parts for *E. craspedia, E. seguieriana *subsp*. seguieriana, E. Fistulosa*, and* E. macroclada* (collected from Malatya) species. But for *E. denticulata, E. eriophora, E. Grisophylla,* and *E. macroclada* (collected from Diyarbakir) species Se amonts are seen highest in the leaf parts. Also Se amountsare observed highest in root parts for *E. alleppica *and* E. Macroclada *(collected from Diyarbakır), and for *E. macroclada *(collected from Trabzon) Se amounts are observed highest in the seed parts differently. In the study of Pehlivanlı *et al.*, the amount of Se has been found as 0,20 ppm in *Euphorbia macroclada* plant; and the World Plant average is defined as 0,49 ppm for Se (31, 33).

Vanadium (V) concentrations of the studied species range from 0.052 to 11.338 mg kg^-1^.As it can be seen from Tables 5, 6 and 7, Vanadium (V) accumulates in root parts mostly for the all species studied. And also it is determinated that the V element accumulates in the roots of studied species are ranked in the order of ESDR > EGVR > EFDR > EMVR > EADR > ECMR > EDKR > EMMR > EEDR > EMDR > EMTR.

In this study, silver (Ag) concentrations of the studied species range from 0.00796 to 2.482 mg kg^-1^. As seen in Table 5-7, Ag accumulates in different parts of the studied species. It is determinated that for *E. craspedia*, *E. eriophora* ve *E. fistulosa*species Ag accumulates more in the root than in the other parts. And for *E. seguieriana *subsp*. seguieriana*, *E. grisophylla* and *E. macroclada *(collected from Diyarbakir and Trabzon) species Ag amounts are found at highest levels in the seed parts. But in *E. denticulata* and *E. Macroclada *(collected from Malatya) species Ag shows more accumulation in branch parts. Also it is observed that Ag accumulates mainly in leaf parts of *E. alleppica *and in the flower parts of *E. macroclada *(collected from Van). 

Cesium (Cs) concentrations of studied species range from 0.004 to 1.237 mg kg^-1^ Cesium (Cs) accumulates in root parts mainly for all species studied. And also it is determinated that the Cs element accumulates in the roots of all species are ranked in the order of EGVR> EMVR > ESDR > EFDR > EMMR > ECMR > EDKR > EADR > EEDR > EMDR > EMTR. In the study of Pehlivanlı *et al.*, the amount of Cs has been found as 5.80 ppm in *Euphorbia macroclada* plant (31).

In this study, rubidium (Rb) concentrations of studied species range from 1.610 to 18.768 mg kg^-1^. As seen in Tables 5, 6, and 7, Rb shows most accumulation in the root parts of the *E. craspedia*, *E. eriophora*, *E. grisophylla*, *E. seguieriana *subsp*. seguieriana*, *E. alleppica*, *E. fistulosa* and *E. macroclada *(collected from Van) species. And it is determinated that for *E. denticulata* and *E. Macroclada *(collected from Malatya and Trabzon) species Rb accumulates more in the flowers than in the other parts. Also it is observed that Rb accumulates most in seed parts for *E. macroclada *(collected from Diyarbakir). In the study of Pehlivanlı *et al.* , the amount of Rv has been found as 41.30 ppm in *Euphorbia macroclada* plant; and the World Plant average is defined as 0.35 ppm for Rb (31, 33).

Strontium (Sr) concentrations of the studied species range from 2.685 to 160.593 ± 21 mg kg^-1^. As seen in Tables 5, 6, and 7, Sr shows most accumulation in root parts of the *E. grisophylla*, *E. Craspedia,* and *E. macroclada *(collected from Van) species. It is determinated that for *E. denticulata*, *E. seguieriana *subsp.* seguieriana* ve *E. fistulosa* species Sr accumulates more in the leafs than the other parts. Also it is observed that Sr accumulated most in the seed parts of *E. alleppica*, *E. Eriophora,* and *E. macroclada *(collected from Malatya, Diyarbakir and Trabzon).

In this study, barium (Ba) concentrations of studied species range from 0.310 to 47.259 mg kg^-1^. Ba amounts are observed highest in the root parts for *E. craspedia*, *E. denticulata*, *E. seguieriana *subsp*. seguieriana*, *E. fistulosa* and *E. macroclada *(collected from Van) species. But for *E. alleppica*, *E. eriophora*, and *E. macroclada* (collected from Diyarbakir, Malatya and Van) species Ba amonts are seen highest in the branch parts. And also it is determinated that Ba accumulates in leaf parts of *E. grisophylla *differently. In the study of Pehlivanlı *et al.*, the amount of Ba has been found as 27.20 ppm in *Euphorbia macroclada* plant, and the World Plant average is defined 29.00 ppm for Ba (31, 33).

As the result when the studied species examined one by one, in *E. craspedia, E. denticulata, E. aleppica, E. grisophylla, E. seguieriana *subsp.* seguieriana, E. fistulosa*, and *E. macroclada *(collected from Malatya, Diyarbakir, and Van) plants metals accumulated in the roots mostly, but in *E. Eriophora* metals accumulated most in the leaf parts, were determinated. And also it was determined that the metal accumulated equally in branch and root for *E. macroclada* collected from Trabzon. The variations in concentrations of several elements are partly due to the differences in anatomy of the specific part of the plant, as well as to the chemical composition of the soil in different localities (43). 

The absorption and accumulation of trace elements in the plant tissue is based on several factors (49). Nevertheless, the absorption and transfering capability of the trace elements may be altered depending on the properties of the element and type of the plant (50).

*Principal Component Analysis (PCA)*

The results of the basic component analysis (PCA) of 12 toxic metals of *Euphorbia* species collected from various regions were given in Table 8 and 9.

As a result of the basic component analysis performed on 12 variables, 5 principle components which eighteen values were greater than 1 were taken into consideration in this data set. According to the PCA result of *Euphorbia* samples, the first 5^th^ principal components explained 74.8% of the total variance. First principal component (PC1) explained 27.0% of the total variance while the second (PC2) 16.6%, the third (PC3) is 12.40%, the fourth 9.70%, and the fifth 9.1%. The variance value steadily decreased in the other basic components. The values given bold in Table 9 were more dominant than the others to explain the basic components. The first principle component explaining 28.30% of the total variance showed the highest variance in the data set. In the PC1, Cr, As, Ni, Pb, Ba, and Co metals were found dominant while in PC2, Ni and Cd (positive direction), and As, Rb and Sr (negative direction), while in PC3, Se, Cd and Sr (positive direction), and Zn (negative direction), and in PC4, Co and Ni (positive direction), Zn and Cd (negative direction). In PC5; however, Cu (positive direction), Zn, and Co (negative direction) were the dominant metals.

The score values of the first five principle components of *Euphorbia* species were given in Table 9. For the first principle component (PC1), Cr, As, Ni, Pb, Ba, and Co metals were in higher concentrations in EGVR, ESDR, EMVR, EFDR, ESDL, ECMR, EDKR, and EADR samples. For the second principle component (PC2) the Ni and Cd concentration were more dominant in EFDR, EFDB, EFDF, ECMF, and ESDF samples while As, Rb, and Sr metals in EDKM, EDKB, EDKL, EDKF, and EDKR samples. For the third principle component (PC3) the Se, Cd, and Sr concentrations were found more dominant in ECMF, EFDF, EFDL, EDKB, EDKL, EFDM, EFDB, and also the samples were found more dominant while Zn in EMTS, EGVR, EMTL and EEDM samples. Accordingly, Co and Ni metals were dominant in EMVL, ESDS, ESDL, ESDF and ESDM sample for the fourth principle component (PC4), and also Zn and Cd metals were dominant in EGVR, EMTB, and EFDF samples. For the fifth principal component (PC5) Cu was more dominant in EFDB and EFDR samples, and Co was more dominant in ESDL and EMTL samples.

**Figure 1:**
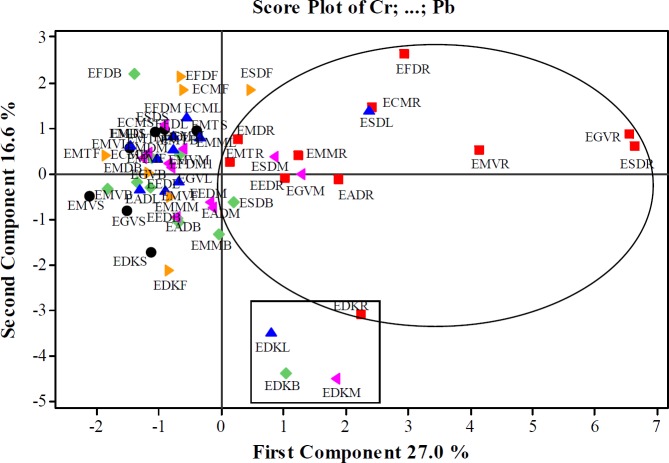
Score plot graphic for PC1 and PC2 in *Euphorbia *samples   seed, ■ root, ♦ branch, ▲ leaf, ►flower, ◄mixed

**Figure 2:**
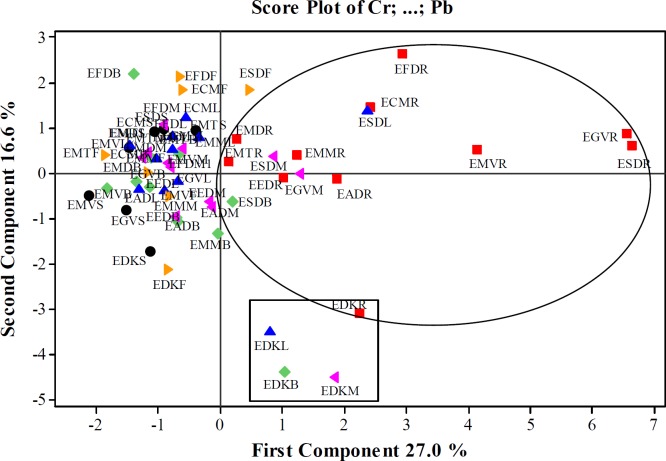
Loading plot for PC1 and PC2 in *Euphorbia* samples.

The score plot and loading plot graphics were given in Figure 1 and 2. It was clearly seen that the amounts of 12 metals exhibited similarity in the root parts of *Euphorbia*species. For this reason, it could be said that the aerial parts and root parts separately fell into two different groups. It was seen that EDKL, EDKM, EDKB, and EDKR samples generated a group in which the samples belong to *E. denticulata* collected from Kayseri. The score and loading graphs showed that Cu, Zn, and Se amounts were lower than those of Cu, Ni, Co, Pb, Cr, Ba, As, Rb, and Sr in the root samples. ESDL, ESDB ESDM, and EGVM samples were similar to root samples in terms of the amounts of Cu, Ni, Co, Pb, Cr, Ba, As, Rb and Sr. These four samples were in the same group with the root samples. Besides, the root samples, all samples except the leaf, branch and mixed parts of *E. denticulata* and ESDF could also be grouped together. Cu, Zn, and Se amounts were closer to each other in these samples.

Different patterns in the trace element contents between different parts of the plants have also been reported in the earlier studies (51,52). Among the factors such as type of soil, forms of metals in the soil, and growth stage of species (51), antagonistic and synergistic interactions between trace elements can influence the absorption and element (53).

*Hierarchical Clustering Analysis (HCA)*

**Figure 3:**
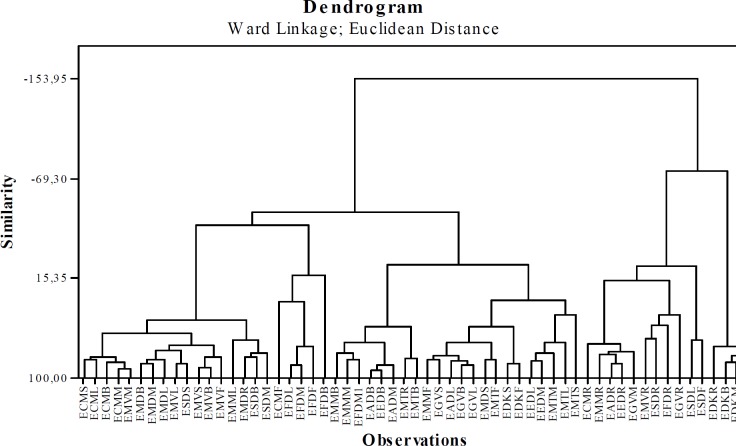
Dendrogram results obtained by Euclidean distance and Ward Linkage method.

Clustering analysis was applied to trace metal concentrations of *Euphorbia* species. The measurements were based on Squared Euclidean distance. The Ward method was used as the classification method. The dendrogram obtained by the Ward method, as shown in the Figure 3. The cluster analysis was performed to compare the distributions of the trace elements (Ag, As, Cd, Co, Cr, Cu, Ni, Pb, Se, Tl, V and Zn ) in the all samples.

It is seen Figure 3, there are 3 different clusters in the dendogram (Similarity=-60)

Cluster 1: EDKL, EDKM, EDKB and EDKR (4)

Cluster 2: ESDR, EGVR, ESDF, ESDL, EMMR, EFDR, EGVM, EEDR, EADR, EMVR and ECMR (11)

Cluster 3: ESDM, ESDB, EMDR, EFDB, EFDF, EFDM, EFDL, ECMF, EDKF, EDKS, EGVB, EGVL, EADL, EGVS, EMMF, EMTS, EMTL, EMTM, EEDM, EEDL, ECM, ECM, ECML and ECMS (44), EMDR, EADM, EADB, EADB, EADB, EMMM, EMMB, EMVF, EMVB, EMVS, ESDS, EMVL, EMDL.

It was seen that the samples in Cluster 1 were the samples belonging to E. *denticulata* collected from Kayseri. But, the EDKF and EDKS which were the parts of this species were grouped in Cluster 3. The samples of the root parts of *Euphorbia* species were mostly grouped in Cluster 2. Thus, the serarating of the aerial parts and roots from each other were easily recognised. The aerial and mixed parts of *Euphorbia* species were mostly located in Cluster 3.

**Table 1:** Herbarium records, gathering places and abbreviations of *Euphorbia *species.

**Plant name**	**Abbreviations**	**Collection location**	**Collection time**	**Herbarium number**	
*E. craspedia seed*	ECMS				
*E. craspedia root*	ECMR				
*E. craspedia branch*	ECMB				
*E. craspedia leaf*	ECML				
*E. craspedia flower*	ECMF				
*E. craspedia mixed*	ECMM	Mardin	June 2015	M. Fırat 31625(VANF)	
*E. denticulata seed*	EDKS				
*E. denticulata root*	EDKR				
*E. denticulata branch*	EDKB				
*E. denticulata leaf*	EDKL				
*E. denticulata flower*	EDKF				
*E. denticulata mixed*	EDKM	Kayseri	June 2015	M. Fırat 31630(VANF)	
*E. aleppica root*	EADR				
*E. aleppica branch*	EADB				
*E. aleppica leaf*	EADL				
*E. aleppica mixed*	EADM	Diyarbakir		M. Fırat 31626(VANF)	
*E. eriophora root*	EEDR				
*E. eriophora branch*	EEDB				
*E. eriophora leaf*	EEDL				
*E. eriophora mixed*	EEDM	Diyarbakir	June 2015	M. Fırat 31627(VANF)	
*E. falcata mixed*	EFDM1	Diyarbakir	June 2015	M. Fırat 31629(VANF)	
*E. grisophylla seed*	EGVS				
*E. grisophylla root*	EGVR				
*E. grisophylla branch*	EGVB				
*E. grisophylla leaf*	EGVL				
*E. grisophylla mixed*	EGVM	Van	June 2015	M. Fırat 30910(VANF)	
*E. seguieriana subsp. seguieriana seed*	ESDS			
*E. seguieriana subsp. seguieriana root*	ESDR			
*E. seguieriana subsp. seguieriana branch*	ESDB			
*E. seguieriana subsp. seguieriana leaf*	ESDL			
*E. seguieriana subsp. seguieriana flower*	ESDF			
*E. seguieriana subsp. seguieriana* mixed	ESDM	Diyarbakır	June 2015	M. Fırat 30905 (VANF)
*E. fistulosa root*	EFDR			
*E. fistulosa branch*	EFDB			
*E. fistulosa leaf*	EFDL			
*E. fistulosa flower*	EFDF			
*E. fistulosa mixed*	EFDM	Diyarbakir	June 2015	M. Fırat 31628(VANF)
*E. macroclada root*	EMMR			
*E. macroclada branch*	EMMB			
*E. macroclada leaf*	EMML			
*E. macroclada flower*	EMMF			
*E. macroclada mixed*	EMMM	Malatya	June 2015	M. Fırat 30906 (VANF)
*E. macroclada seed*	EMDS			
*E. macroclada root*	EMDR			
*E. macroclada branch*	EMDB			
*E. macroclada leaf*	EMDL			
*E. macroclada mixed*	EMDM	Diyarbakir	June 2015	M. Fırat 30906 (VANF)
*E. macroclada seed*	EMVS			
*E. macroclada root*	EMVR			
*E. macroclada branch*	EMVB			
*E. macroclada leaf*	EMVL			
*E. macroclada flower*	EMVF			
*E. macroclada mixed*	EMVM	Van	June 2015	M. Fırat 30906 (VANF)
*E. macroclada seed*	EMTS			
*E. macroclada root*	EMTR			
*E. macroclada branch*	EMTB			
*E. macroclada leaf*	EMTL			
*E. macroclada flower*	EMTF			
*E. macroclada mixed*	EMTM	Trabzon	June 2015	M. Fırat 30906 (VANF)

**Table 2:** Optimal ICP-MS operating conditions for analysis of samples

**Instrument parameter**	**Condition**
RF power	1550 W
RF frequency	27.12 MHz
RF Matching	1.80 V
Carrier gas (inner)	1.1 L/min
Makeup Gas	0.9 L/min
Plasma gas	Ar X50S 5.0
Plasma gas flow (Ar)	15 L/min
Nebulizer pump	0.1 rps
Sample intake	0.5 mL/min
Spray chamber temperature	2 °C
Resolution m/z	244 amu
Background	<5 cps (9 amu)
Short-term stability	<3% RSD
Long-term stability	<4% RSD/2 h
Isotopes measured	51V, 52Cr, 59Co, 60Ni, 63Cu, 66Zn, 75As, 78Se, 107Ag, 111Cd, 205Tl, 208Pb.

**Table 3:** Analytical parameters of the ICP-MS method.

**Elements**	**Linear range (µgkg** **-1** **)**	**Regression**	**R**	**Limit of detection (µg kg** **-1** **)**	**Limit of quantification (µg kg** **-1** **)**
Ag	0-100	y= 0.1202 x + 0.0013	0.9998	0.0066	0.0198
As	0-100	y= 0.0429x + 0.0012	0.9999	0.0088	0.0264
Ba	0-100	y= 0.0092 x + 0.0005	0.9999	0.0126	0.0378
Cd	0-100	y= 0.0069 x + 0.0005	0.9999	0.0042	0.0126
Co	0-100	y= 0.0661 x + 0.0012	0.9999	0.0196	0.0588
Cr	0-100	y= 0.0573 x + 0.0170	0.9997	0.0461	0.1383
Cs	0-100	y= 0.0710 x + 0.0002	0.9997	0.0041	0.0123
Cu	0-100	y= 0.7185 x + 0.8796	0.9998	0.0572	0.1716
Ni	0-100	y= 0.0148 x + 0.0316	0.9998	0.5303	1.5909
Pb	0-100	y= 0.0760 x + 0.0103	0.9998	0.0268	0.0804
Rb	0-100	y= 0.2538 x + 0.0020	0.9999	0.0048	0.0144
Se	0-100	y= 0.0026 x + 0.0019	0.9997	0.0765	0.2295
Sr	0-100	y= 0.3451 x + 0.1645	0.9999	0.0668	0.2004
Tl	0-100	y= 0.0571 x + 0.0004	0.9998	0.0205	0.0615
V	0-100	y= 0.0630 x + 0.0018	0.9998	0.0025	0.0075
Zn	0-100	y= 0.0435 x + 0.1530	0.9995	0.5737	1.7211

**Table 4:** Accuracy assessment of analysis of Certified Reference Material Tomato Leaves (CRM NIST1573a)a.

**Elements**	**Certified (mg kg** **-1** **)**	**Found (mg kg** **-1** **)**	**Recovery (%)**
Cd	1.52±0.04	1.50±0.11	98.02
Co	0.57±0.02	0.58±0.01	98.24
Cr	1.99±0.06	2.03±0.03	102.5
Cu	4.70±0.14	4.68±0.08	99.57
Ni	1.59±0.07	1.61±0.03	101.89
Zn	30.9±0.7	32.10±0.20	103.89

aValues expressed are means ± S.D. of three parallel measurements. Values with different letters in the same column were significantly different with Student’s t-test (*p*< 0.05).

**Table 5:** Concentration of metal in dry mass in *E. craspedia, E. denticulata, E. aleppica *and *E. eriophora *species(µg kg-1± Standard deviation).

**Sample**	**Ag**	**As**	**Ba**	**Cd**	**Co**	**Cr**	**Cs**	**Cu**	**Ni**	**Pb**	**Rb**	**Se**	**Sr**	**Tl**	**V**	**Zn**
ECMS	838±8	52±2	1976±7	124±1	174±2	1547±11	16.79±1.16	7167±14	5571±12	483±5	5551±15	816±10	10176±12	2.54±0.05	503±3	24194±12
ECMR	1041±18	413±3	22475±14	451±3	955±5	7780±8	227±6	6288±12	8038±11	1596±12	7999±13	667±11	38652±18	39.84±0.36	4614±6	20347±7
ECMB	919±11	60±2	9327±13	320±2	109±5	917±7	18.71±0.35	2607±7	2188±8	533±5	6565±12	949±13	36800±20	8.54±0.12	414±5	18477±8
ECML	876±10	137±2	3250±10	262±2	626±7	2353±18	37.96±1.40	10423±8	3638±10	1028±5	4128±10	773±11	20946±15	5.51±0.09	1185±7	14200±13
ECMF	3869±15	111±4	4146±14	244±3	551±9	1601±10	36.45±1.00	3517±10	3937±10	2045±11	3833±14	2430±3	16624±17	3.92±0.03	1151±9	14590±15
ECMM	768±8	153±3	6547±12	228±2	439±5	1887±8	50.11±1.10	3304±4	3703±12	753±10	6209±18	826±3	26952±16	10.16±0.13	1300±13	19633±20
EDKS	904±4	91±2	2242±16	27.71±0.49	106±1	340±5	17.30±1.50	6837±7	617±2	924±1	13426±10	747±3	131646±18	0.60±0.02	121±3	29196±12
EDKR	593±3	943±3	33268±14	126±1	362±1	5744±2	193±2	4784±6	1460±7	878±1	9978±12	533±3	264496±11	20.81±1.10	4584±10	17486±10
EDKB	812±2	854±3	18163±15	62±1	121±1	711±2	34.11±0.36	3042±5	344±2	483±1	16934±12	732±2	285422±12	2.14±0.04	270±3	13144±11
EDKL	768±3	752±2	11097±17	66±3	285±1	410±2	43.44±044	2965±2	352±2	1461±1	11441±17	864±4	298418±13	1.66±0.10	415±4	20451±18
EDKF	726±6	157±2	2407±18	53±2	195±3	435±1	28.78±0.72	5099±5	697±5	582±1	18768±16	754±4	101924±10	3.57±0.20	244±2	29354±11
EDKM	817±7	841±2	24906±12	59±1	309±2	2886±7	129±1	4871±2	960±1	731±1	18828±18	731±2	325285±31	8.52±0.52	1473±13	25175±16
EADR	908±8	246±3	39309±27	110±2	796±5	8461±11	145±2	5063±12	5577±14	1086±6	14126±18	635±3	29084±23	20.26±0.12	4620±17	29189±21
EADB	611±7	93±2	42512±42	50.84±2.47	121±3	1061±8	12.21±0.43	7074±15	1014±15	409±3	9016±15	307±3	38449±34	4.82±0.08	428±7	22146±17
EADL	1092±10	75±1	20457±30	48.60±1.72	384±4	699±7	14.34±0.12	8018±17	1092±11	215±2	8305±18	558±3	21738±30	1.49±0.01	574±9	34603±16
EADM	713±9	131±2	45127±17	66±1	442±5	2399±10	45.53±0.26	4588±9	1970±16	408±4	9474±15	505±3	38574±47	6.82±0.05	1683±16	27717±12
EEDR	796±4	219±4	27034±14	134±2	595±7	7436±5	98±1	4584±4	4419±5	665±5	12289±35	647±2	21729±29	13.28±0.12	3559±12	19053±5
EEDB	681±3	108±3	35598±19	63±1	153±6	1846±7	17.84±1.12	3819±6	1116±12	175±4	9993±38	355±3	33412±17	3.56±0.08	632±8	21520±9
EEDL	706±5	141±3	25554±18	82±2	387±3	1130±4	23.49±0.90	6623±6	1308±11	526±5	8995±30	687±7	29751±25	6.54±0.40	764±9	57363±10
EEDM	1210±12	109±2	34625±48	69±1	474±4	2461±6	42.57±1.23	5533±8	2314±14	774±6	10744±40	330±2	33652±18	7.80±0.33	1597±17	46703±11
EFDM1	939±10	65±2	33585±21	125±2	479±3	1446±7	17±0.21	4233±9	1776±12	521±6	3949±21	910±5	49458±20	4.09±0.02	809±7	27614±

Measured value = mean ± SD. Each sample was measured for three times

**Table 6:** Concentration of metal in dry mass in *E. grisophylla, E. seguieriana *subsp*. seguieriana *and *E. fistulosa *species(µg kg-1± Standard deviation).

**Sample**	**Ag**	**As**	**Ba**	**Cd**	**Co**	**Cr**	**Cs**	**Cu**	**Ni**	**Pb**	**Rb**	**Se**	**Sr**	**Tl**	**V**	**Zn**
EGVS	1842±19	46,14±0,60	1334±18	14.42±0.90	245±2	483±1	33.18±1.99	8635±17	1774±2	185±3	14343±12	504±3	13212±18	1.80±0.04	354±8	37368±16
EGVR	1018±18	478±1	64321±36	479±6	1698±7	11985±10	1237±16	4307±13	8498±9	6813±12	18325±11	444±2	64648±15	159±5	10092±32	40831±21
EGVB	1046±17	96±1	5035±21	163±3	253±2	1588±12	47.94±1.24	6226±11	1242±4	544±4	8838±19	526±6	40981±11	10.12±0.45	966±12	28427±12
EGVL	735±6	164±2	6519±23	39.31±0.63	555±4	2679±8	114±2	4229±9	1943±11	1065±8	8404±10	526±7	15176±17	16.81±0.21	2222±34	28506±13
EGVM	677±5	460±4	16617±17	152±4	924±6	5053±8	328±6	4248±8	3877±6	2054±7	10216±15	601±5	27005±23	41.46±0.32	4289±45	31608±14
ESDS	2482±20	41.42±3.39	2542±14	30.96±0.15	1377±19	395±11	9.48±0.58	5281±19	4884±21	443±3	3557±27	679±4	27016±24	28.57±1.05	170±4	21283±54
ESDR	870±17	1203±7	47259±45	369±3	1699±17	12195±27	553±6	3925±18	12209±41	3801±17	9999±27	762±7	122883±30	67±3	11338±43	24699±65
ESDB	721±15	364±4	19740±35	143±2	527±12	2110±17	44.19±1.35	2311±19	2637±16	1165±23	4403±12	493±5	106391±32	7.99±0.54	1380±16	11438±33
ESDL	798±16	608±13	16301±15	302±5	3861±28	1623±20	57.02±1.57	3501±39	7202±23	352±8	3651±19	959±1	133426±39	11.49±0.17	1443±13	29302±62
ESDF	1507±19	78±3	4419±16	68±1	2757±31	478±12	14.59±0.81	5236±20	8937±34	445±9	3960±10	554±4	46474±28	17.06±0.34	304±5	25608±16
ESDM	816±11	317±7	15310±18	169±3	1774±17	1826±13	52.46±1.33	3246±28	4716±19	1375±14	3924±11	360±3	94692±75	9.65±0.09	1549±20	14932±45
EFDR	30.53±1.88	318±4	18811±45	301±2	1095±16	12913±17	317±4	21195±11	10248±34	1660±18	5569±17	564±5	36919±30	32.44±1.40	7507±34	19154±22
EFDB	17.68±1.12	66±1	7247±16	270±1	155±3	2622±14	22.30±0.79	44488±45	2365±17	291±8	2685±15	812±6	36889±18	5.17±0.12	961±12	10348±13
EFDL	8.40±0.29	120±2	9612±17	561±4	143±2	1808±15	32.45±0.85	6297±24	1598±18	380±7	3573±24	985±6	92236±50	6.76±0.23	1031±25	29090±16
EFDF	11.01±0.45	66±1	7483±13	827±11	166±4	1232±12	21.07±0.73	6204±38	3457±34	241±6	3941±19	1321±12	41631±38	3.11±0.04	724±14	36801±18
EFDM	7.96±0.07	64±1	7573±10	539±5	161±2	2158±28	289±7	3870±16	2160±18	265±6	3546±25	868±9	46827±32	4.51±0.06	981±15	17404±19

Measured value = mean ± SD. Each sample was measured for three times

**Table 7:** Concentration of metal in dry mass in *E. craspedia, E. denticulata, E. aleppica *and *E. eriophora *species(µg kg-1± Standard deviation).

**Sample**	**Ag**	**As**	**Ba**	**Cd**	**Co**	**Cr**	**Cs**	**Cu**	**Ni**	**Pb**	**Rb**	**Se**	**Sr**	**Tl**	**V**	**Zn**
EMMR	795±6	135±4	22250±22	252±2	680±7	9781±27	308±5	3356±18	3606±18	1079±8	8047±27	423±2	54122±39	42.49±1.84	3863±12	20433±42
EMMB	968±12	37.52±1.25	52595±50	210±1	303±6	572±10	20.73±1.08	3379±22	1490±22	482±7	8056±34	605±4	160593±21	6.20±0.04	348±3	22866±48
EMML	753±15	52.71±1.10	7888±33	96±1	224±4	1278±14	24.42±0.37	1895±20	9866±36	283±3	3118±10	528±4	81623±25	4.54±0.08	426±7	25723±40
EMMF	876±16	60±1	3191±19	157±2	175±2	2064±17	44.49±1.01	7768±23	2337±19	394±4	10348±18	847±7	27714±32	3.41±0.04	664±8	39102±34
EMMM	897±9	62±1	25107±25	167±3	177±3	1250±15	49.85±1.90	3707±13	1513±12	373±5	9922±16	688±4	95761±35	7.93±0.15	757±9	30760±31
EMDS	1102±10	33.55±0.75	1904±17	37.46±0.72	417±2	255±1	7.40±0.07	5558±15	5076±5	99±1	5074±10	448±3	9294±16	1.84±0.02	172±1	38362±10
EMDR	815±13	75±1	22601±25	110±2	642±4	5380±5	35.84±0.40	3583±13	4847±5	1107±12	2972±10	384±3	33413±12	8.69±0.03	2875±5	12331±13
EMDB	1029±10	35.02±0.20	22953±23	62±1	190±1	866±4	9.31±0.64	2297±10	2046±4	131±2	3609±21	457±4	35274±17	2.11±0.02	416±2	16570±20
EMDL	899±14	60±2	9475±17	79±1	820±3	1009±5	10.91±0.98	2833±12	2579±4	1634±16	2983±20	472±2	28135±27	1.70±0.06	517±3	18703±21
EMDM	800±9	58±1	12028±15	69±1	481±2	1161±6	13.46±0.62	3161±16	2878±3	402±5	3456±15	764±3	28085±26	1.98±0.06	716±1	16789±27
EMVS	866±16	11.04±0.96	310±1	5.14±0.06	156±1	213±2	15.66±1.05	4130±13	604±3	71±2	8009±12	608±5	3449±14	1.76±0.02	52±1	12107±33
EMVR	888±11	463±3	42541±15	99±1	1363±16	12329±20	673±4	4038±22	9215±12	2842±19	12226±27	545±4	53139±20	79±2	6807±12	21604±19
EMVB	867±17	43.02±1.04	4298±17	14.23±0.92	124±1	755±7	26.76±0.75	2555±16	1280±13	278±4	5026±14	707±7	32691±10	6.29±0.07	300±1	9786±16
EMVL	758±12	112±2	2865±17	14.81±0.02	1188±14	1270±20	51.02±1.27	3165±13	2553±17	380±5	5730±20	831±8	21641±27	8.83±0.50	807±9	13438±24
EMVF	1567±18	110±1	4250±12	22.23±0.98	540±8	1451±12	66±2	3937±18	2579±16	738±7	12080±14	719±5	19196±18	8.10±0.38	1180±14	16823±15
EMVM	857±11	119±4	4021±12	172±3	491±3	1493±10	49.33±1.14	3668±10	2376±10	966±8	7106±12	730±6	22044±20	6.38±0.44	777±11	12602±17
EMTS	1401±14	28.65±1.34	5223±41	68±1	322±4	444±3	3.64±0.02	9640±17	1169±23	5396±18	1513±19	264±4	23523±56	2.02±0.06	76±1	45969±26
EMTR	833±13	370±2	53541±23	269±8	268±3	1982±12	31.27±2.19	8046±24	948±16	1046±21	1831±18	748±5	28953±50	7.79±0.41	949±6	26948±27
EMTB	1006±12	71±1	53635±32	328±5	124±2	728±7	9.59±0.19	2696±13	825±18	729±9	1614±17	909±16	36533±45	3.36±0.12	308±3	38951±45
EMTL	789±6	104±2	13716±25	84±2	931±5	576±6	15.80±1.23	4004±17	742±4	684±7	1610±13	654±11	27175±48	12.81±1.12	496±4	79547±58
EMTF	753±7	40.03±1.53	2106±24	35.54±2.32	362±3	229±3	4.49±0.25	9172±15	1458±12	797±5	2923±21	263±9	10798±47	4.38±0.23	101±2	43875±45
EMTM	886±9	144±4	20895±48	125±2	620±4	753±6	14.68±1.55	4759±20	794±7	928±8	2002±14	715±11	21413±49	9.48±0.73	451±2	48516±42

Measured value = mean ± SD. Each sample was measured for three times

**Table 8:** The loading, eigenvalue, variance and cumulative variance values of the principle components of the *Euphorbia *species

**Heavy Metal**	**PC1**	**PC2**	**PC3**	**PC4**	**PC5**
As	**0.424**	-0.303	0.215	0.053	-0.047
Ba	**0.320**	-0.110	-0.231	-0.199	-0.015
Cd	0.180	**0.323**	**0.385**	-0.486	-0.053
Co	**0.297**	0.252	-0.033	**0.442**	-0.363
Cr	**0.468**	0.149	-0.081	-0.039	0.274
Cu	-0.036	0.220	0.070	-0.112	**0.741**
Ni	**0.378**	**0.372**	0.009	**0.316**	-0.010
Pb	**0.374**	0.118	-0.197	-0.133	-0.027
Rb	0.220	-0.464	-0.074	-0.041	0.170
Se	-0.049	0.138	**0.633**	-0.235	-0.253
Sr	0.203	-0.520	**0.312**	0.024	-0.050
Zn	-0.040	0.043	-0.446	-0.453	-0.377
Eigenvalue	3.232	1.9892	1.4916	1.1674	1.0936
Variance (%)	27.0	16.6	12.4	9.7	9.1
Cumulative (%)	27.0	43.6	56.0	65.7	74.8

**Table 9:** The scores of the first fifth rotated principal component.

**Kodu**		**PC1**	**PC2**	**PC3**	**PC4**	**PC5**
ECMS		-1,08944	0,93105	0,32039	0,48762	0,29069
ECMR		**2,41368**	1,47056	0,65383	-0,12459	0,34631
ECMB		-1,11903	0,34812	1,21259	-0,51661	-0,28381
ECML		-0,56416	1,24330	0,93665	0,37789	0,72276
ECMF		-0,63444	**1,85604**	**4,00909**	-0,92011	-1,56341
ECMM		-0,60226	0,54319	0,71380	0,22811	-0,22925
EMMR		1,22857	0,41042	-0,48833	0,05543	0,62407
EMMB		-0,05081	-1,33690	-0,01544	-1,02073	-0,19229
EMML		-0,34863	0,79820	-0,13735	1,29773	-0,37893
EMMF		-1,18077	0,03874	-0,06839	-0,59397	0,10958
EMMM		-0,72146	-0,93773	-0,11456	-0,69615	-0,17629
EDKS		-1,13520	-1,70727	0,14008	-0,02826	0,33831
EDKR		**2,24577**	-3,09580	1,09450	0,01850	0,46556
EDKB		1,03300	-4,37114	**1,79086**	0,37042	0,23963
EDKL		0,80126	-3,50243	**1,81457**	0,14587	-0,42195
EDKF		-0,85605	-2,11008	0,07840	-0,00808	0,27586
EDKM		1,84236	-4,50690	1,31887	-0,11851	0,22431
EADR		**1,86918**	-0,12513	-1,00388	-0,14563	0,48384
EADB		-0,70584	-0,98302	-1,29084	-0,32161	0,85355
EADL		-1,31922	-0,36237	-0,93845	-0,23092	0,23948
EADM		-0,12770	-0,74031	-1,12513	-0,42469	0,14747
EEDR		1,01501	-0,08578	-0,32353	0,25719	0,69649
EEDB		-0,69238	-1,05687	-1,08712	-0,09434	0,51404
EEDL		-0,91438	-0,37366	-1,45830	-1,35211	-0,69535
EEDM		-0,17316	-0,61418	-2,10401	-0,76382	-0,10828
EMDS		-1,48346	0,58714	-1,08796	0,74766	-0,23983
EMDR		0,24728	0,77762	-0,58283	0,97966	0,46181
EMDB		-1,36276	-0,19147	-0,46443	0,52303	0,06727
EMDL		-0,78236	0,51523	-0,55443	0,92319	-0,32301
EMDM		-1,24542	0,36477	0,26470	0,74297	-0,22213
EFDM1		-0,80062	0,13991	0,09388	-0,59595	-0,53746
EMVS		-2,12713	-0,46079	-0,00190	1,01568	0,50853
EMVR		**4,13601**	0,53252	-1,09271	0,68137	0,48111
EMVB		-1,82830	-0,31534	0,38780	0,98746	0,20755
EMVL		-1,02587	0,31523	0,46353	**1,61179**	-0,44463
EMVF		-0,83797	-0,51261	-0,02345	0,96092	0,23850
EMVM	-0,84989		0,22748	0,56923	0,61065	0,10662
EGVS	-1,52681		-0,78779	-1,09120	0,18268	0,60221
EGVR	**6,55251**		0,87546	-2,06512	-2,16822	-0,19930
EGVB	-1,15220		-0,30525	-0,29530	-0,06791	0,33755
EGVL	-0,68555		-0,18138	-0,81384	0,49581	0,02365
EGVM	1,29212		0,00513	-0,60372	0,06313	-0,20365
ESDS	-0,93839		0,99776	-0,04558	**1,76176**	-0,54602
ESDR	**6,63208**		0,61333	0,61877	0,07122	-0,38952
ESDB	0,18580		-0,62551	0,38014	0,69178	-0,01857
ESDL	**2,37292**		1,38013	1,49202	**2,20466**	-2,76750
ESDF	0,45086		**1,85112**	-0,34050	**2,92542**	-1,35264
ESDM	0,86052		0,38491	-0,04225	**1,69351**	-0,61354
EMTS	-0,41017		0,96887	-2,43334	-0,98815	0,00432
EMTR	0,13322		0,25709	-0,00104	-1,67877	0,05877
EMTB	-0,66671		0,45027	-0,13153	-2,40858	-1,09765
EMTL	-1,47625		0,60411	-2,12158	-1,45725	-2,25223
EMTF	-1,87295		0,42143	-1,70065	0,08700	0,14662
EMTM	-1,16779		0,46994	-0,93536	-0,95370	-1,12297
EFDR	**2,92025**		**2,64965**	0,16908	0,52849	**2,66020**
EFDB	-1,39646		**2,21735**	**1,68715**	-0,61094	**5,33509**
EFDL	-0,78134		0,81365	**1,87618**	-1,70087	-0,34305
EFDF	-0,67772		**2,14691**	**2,74089**	-2,76698	-0,95028
EFDM	-0,90133		1,08309	**1,75703**	-0,97115	-0,13821

## Conclusion

When the studied *Euphorbia *species were compared in terms of their metal contents; V, Tl, Cr, and Ni metals in *E. eriophora* species, Ba in *E. aleppica *species, As and Co metals in *E. seguieriana *subsp.* seguieriana*species, Ag and Se metals in *E. craspedia *species, Cu and Cd metals in *E. fistulosa *species, Cs and Pb metals in *E. grisophylla *species, Zn in *E. macroclada* species (collected from Trabzon) and also Rb and Sr metals in *E. denticulata *species was determinated higher.

Some metal elements like Cr, As, Cd, V, and Cs accumulated most in the root parts almost all of the studied *Euphoria *species were observed. But for some metals like Tl, Pb, Ni, and Co although they generally accumulated in the root parts, some exceptions were observed. For instance Tl amounts found highest in root parts of the all studied *Euphoria* species except *E. macroclada* species (collected from Trabzon). And another metal element Pb amounts were measured highest in the root parts of the all studied *Euphoria* species except *E. denticulata *and *E. Macroclada *(collected from Diyarbakır and Trabzon). Additionally, it was observed that Ni accumulated most in roots among the studied parts except EMML, EMDS, and EMTS. And also Co amounts were found highest in the roots among the studied parts except all of the *E. seguieriana *subsp.* seguieriana *parts, EMDL and EMTL. It was observed that Cu, Zn, Rb, Sr, and Ba metals accumulated in leaves, flowers, branches, seeds and roots differently.

As a summary, it can be predicted that these species will be used as ornamental plants in landscape architecture due to both their toxic metals retention properties and their beautiful appearance.

Nine different *Euphorbia *species collecting from Diyarbakir, Malatya, Trabzon, Kayseri, Mardin and Van were divided into their parts; such as, flowers, seeds, roots, branches, and leaves. Accordingly, 11 species and totally 59 parts were studied for their metal contents.

As a result of the PCA analysis, it was seen that the aerial parts and the roots of the species were separated from each other (the score graph in Figure.1). The aerial parts of the species generated a separate cluster. Similarly, EDKL, EDKM, EDKB, and EDKR samples belonging to *E. denticulata* collected from Kayseri also formed a separate group.

The results of HCA analysis were found to be parallel to those of PCA analysis. In addition, HCA and PCA not only showed the seperation of aerial parts and roots of the species, but also indicated that the flowers, mixed, root, and branches parts of *E. denticulata* belong to a different cluster member. 
